# “Stuck in the Middle with You”: intermediate cell states are not always in transition

**DOI:** 10.1172/JCI174633

**Published:** 2023-11-15

**Authors:** Jennifer M.S. Sucre, A. Scott McCall, Jonathan A. Kropski

**Affiliations:** 1 Department of Pediatrics, Vanderbilt University School of Medicine, Nashville, Tennessee, USA.; 2 Department of Cell and Developmental Biology, Vanderbilt University, Nashville, Tennessee, USA.; 3 Department of Medicine, Vanderbilt University School of Medicine, Nashville, Tennessee, USA.

## Abstract

The era of single-cell multiomics has led to the identification of lung epithelial cells with features of both alveolar type 1 (AT1) and alveolar type 2 (AT2) pneumocytes, leading many to infer that these cells are a distinct cell type in the process of transitioning between AT2 and AT1 cells. In this issue of the *JCI*, Wang and colleagues demonstrated that many so-called “transitional cells” do not actually contribute to functional repair. The findings warrant a reimagining of these cells as existing in a nondirectional, intermediate cell state, rather than moving through a transitory process from one cell type to another. We look forward to further exploration of diverse cell state expression profiles and a more refined examination of hallmark gene function beyond population labeling.

The advent of single-cell transcriptomics has provided biologists an unprecedented opportunity to capture previously unappreciated cell states. With the discovery of different cell states has come an imperative to name and characterize the function of these cell states. In the lung, many of these cell states appear during periods of local or organ-wide transition—both during organogenesis and in the attempt to repair the lung after injury. In this new single-cell transcriptomics world, there has been a rapid increase in manuscripts reporting the discovery and naming of these in-between states (1–5). Indeed, this major technical innovation has heralded a return to the ancient science of taxonomy and a sense that the state of the field is, as the song “Stuck in the Middle with You” by Stealers Wheel aptly describes, “all over the place” as we try to make “sense of it all” (6) by naming, and occasionally renaming, previously unappreciated cell types.

Over time, the approach used to describe, name, and characterize these intermediate states has evolved. In the alveolar space, single-cell RNA-Seq studies have consistently identified cells with transcriptomic signatures that contain hallmark genes of both alveolar type 1 (AT1) and alveolar type 2 (AT2) cells, the predominant epithelial cells in this niche (5, 7–9). With some exceptions (10), the prevailing model of lung development and lung repair in the adult suggests that some AT2 cells serve as a reservoir of progenitors in the lung, differentiating into AT1 cells during normal development and in response to injury (11, 12). Upon finding experimentally emergent cells with features of both AT1 and AT2 cells, researchers intuitively inferred that these intermediate cell states were in the process of transitioning from AT2 cells to AT1 cells. Furthermore, although single-cell transcriptomics provides a comprehensive expression profile for every cluster of cells, as characterization of these cell states has evolved, we have collapsed this expansive profile to include one or two hallmark genes (e.g., *Krt8*, *Cdkn1a*). While this shorthand allows for ready comparison and communication about these intermediate cell states, the use of a limited number of genes as apparent markers to find commonality from across data sets has resulted in a perhaps overly convergent conceptualization of these cells, narrowing our ability to contrast differences between these populations. This loss of nuance from premature closure in hopes to identify a cell type has led to an underappreciation of the diversity of function and potential roles in pathology and resilience for these intermediate cell states.

In this issue of the *JCI*, pioneering work by Wang and co-authors demonstrates a compelling role of intermediate states in the progression or resolution of fibrosis using mouse models and human samples (13). A standard part of any single-cell sequencing analysis is to group and label cell populations as known cell types on the basis of established hallmark genes. While necessary, this tool can create the misapprehension that hallmark genes are merely widely reported markers of identification, preventing us from more deeply interrogating their role and mechanistic function in physiology and pathology. An additional danger in this approach is to assume that any cell expressing a hallmark gene will also share the behavior and features of cells with a particular identity. Overturning these assumptions, Wang et al. delve deeply into the possible functions of one hallmark gene that corresponds with an intermediate cell state, keratin 8 (*Krt8*) (13). While keratins in general have been described as epithelial cell filaments conferring mechanical stability (14), Wang et al. (13) explore some of the reported but not well-characterized keratin functions in activating inflammation and regulating cell differentiation (12, 15). Building on their surprising finding that upon targeted analysis, SNPs in the *KRT8* locus (but no other keratins) may be associated with a risk for pulmonary fibrosis (PF) in humans, the authors explore the time course of KRT8 expression in multiple injury models. They found that KRT8 expression preceded the development of fibrosis and resulted in the expansion of an intermediate cell population. Furthermore, mice lacking KRT8 appeared to be partly protected from fibrosis in classic injury models. Taken together, their data demonstrate that many of the intermediate cell states observed, especially those with high KRT8 expression, were not transitioning between AT2 and AT1 cell states, but actually discontinued epithelial repair and participated in fibrosis. While we and many others have used the term “transitional” to describe cell states with features of AT2 and AT1, we propose that the classification of “transitional cell types,” which implies directionality between AT2 and AT1, could be reinterpreted on the basis of the findings of Wang et al. as well as of human organoid data suggesting that some of these intermediate cell states lead to fibrosis or even precancerous disease (16).

The nomenclature used to define the cellular architecture of the lung during homeostasis and injury repair provides both model and scaffold upon which our scientific questions are based, with the use of the term “transitional” implying a single directionality from AT2 to AT1. Indeed, some have even proposed intratracheal installation of these cells as a cure for chronic lung disease. Work shown in Wang et al. (13) and studies by others suggest that additional complexity is required in our understanding of these cells and of intermediate cell states. We propose moving away from thinking about presumed transitional cell types as defined populations having a distinct identity and instead toward embracing a concept of intermediate cell states to describe transient cell clusters with features beyond their adjacent classically defined cell types. This approach doesn’t bias our thinking toward assuming that all intermediate cell types with common hallmark gene expression are in fact the same cell state and allows for free interrogation of the plasticity and nuance of these cell populations within a physiologic context. Furthermore, additional exploration of intermediate cell states in a variety of different injury conditions and models (in humans and mice) may reveal mechanisms that promote repair as opposed to those that lead to permanent, progressive injury and scarring. A nuanced approach in interpreting single-cell multiomics allows us to discern which intermediate cell states to target therapeutically and to separate the cells acting as “clowns to the left” and “jokers to the right”(6) to help the cells stuck in intermediate states “get down the stairs” (6) to promote repair after injury. The work by Wang et al. (13) advances our understanding of repair after lung injury in general. By reminding us that hallmark genes have roles beyond merely providing markers for the purposes of identification, this manuscript provides a foundation for future studies that interrogate the role of hallmark genes in cellular and organ function, particularly in cell states that emerge during times of great structural transition such as development and injury repair.

## Version 1 11/15/2023

Electronic publication
